# *TNF* genetic polymorphism (rs1799964) may modify the effect of the dietary inflammatory index on gastric cancer in a case–control study

**DOI:** 10.1038/s41598-020-71433-9

**Published:** 2020-09-03

**Authors:** Jeeeun Kim, Jeonghee Lee, Il Ju Choi, Young-Il Kim, Joohon Sung, Jeongseon Kim

**Affiliations:** 1 Department of Cancer Biomedical Science, Graduate School of Cancer Science and Policy, National Cancer Center, Goyang-si, Gyeonggi-do 10408 South Korea; 2 Division of Genome and Health Big Data, Department of Public Health Sciences Graduate School of Public Health, Seoul National University, Seoul, South Korea; 3 Center for Gastric Cancer, National Cancer Center, Goyang-si, South Korea

**Keywords:** Cancer epidemiology, Gastric cancer

## Abstract

The inflammatory process is known to increase the risk of gastric carcinogenesis, and both genetic and dietary factors are associated with inflammation. In the present study of 1,125 participants (373 cases and 752 controls), we determined whether the dietary inflammatory index (DII) is associated with the risk of gastric cancer (GC) and investigated whether a *TNF* polymorphism (rs1799964) modifies this association. Semi-quantitative food frequency questionnaire derived data were used to calculate the DII scores. Odds ratios (OR) and 95% confidence intervals (CI) were calculated using multivariable logistic models adjusted for confounders. When we stratified the data by sex, the association between GC and the DII was significant only among the women (OR, 2.27; 95% CI 1.25–4.19), and the DII effect on the risk of GC differed depending on the *TNF* genotype (OR, 2.30; 95% CI 1.27–4.24 in TT genotype; OR, 0.78; 95% CI 0.37–1.65 in CC + CT, p for interaction = 0.035). Furthermore, the association between the DII and GC was significant in the *Helicobacter pylori*-positive group; similarly, the effect differed based on the *TNF* genotype (OR, 1.76; 95% CI 1.13–2.73 in TT genotype; OR,0.98; 95% CI 0.54–1.77 in CT + CC, p for interaction = 0.034). In conclusion, rs1799964 may modify the effect of the DII on GC.

## Introduction

According to the GLOBOCAN results reported in 2018, gastric cancer (GC) is the fifth most common cancer worldwide, and the highest mortality rates are found in East Asia, including Korea^1^. In 2016, the incidence of GC was first and second among all types of cancer in Korea^2^. Such a high prevalence of GC may be attributed to *Helicobacter pylori* infection and chronic inflammation caused by chronic gastritis^3^.

The inflammatory process is caused by cytokine secretion and increases the risk of the development of gastric carcinogenesis^4,5^. Among these cytokines, tumor necrosis factor (*TNF*) is among the most important factors inhibiting gastric acid secretion, causing the development of GC^6^. *TNF* production is generally regulated at the transcriptional level^6^. Polymorphisms located in the promoter region of the *TNF* gene affect the level of *TNF*. Indeed, genetic polymorphisms of the *TNF* gene have been proposed as candidate risk factors for GC^7^. However, the results of studies investigating the association between *TNF* genetic polymorphism and GC risk have been inconsistent^8,9^. These results indicate the need for further studies considering other factors, such as individual lifestyle factors.

Environmental factors and host genetic factors also contribute to the chronic inflammatory response. Among these factors, dietary components contain both carcinogens and anticancer substances and are known to control the risk of cancer^10^. For example, the consumption of fruits, vegetables, and beans containing antioxidants has been reported to help reduce inflammation, whereas the consumption of saturated fats, refined carbohydrates, and processed meat can cause inflammation^11,12^. However, the analysis of a single food group or single nutrient does not cover the entire intake of an individual, and there is a limit to identifying the association with the disease^13^. Since the results of most studies focusing on single nutrients are inconsistent, it is necessary to assess the overall effect of diet-induced inflammation reflecting the combination of nutrients consumed by individuals on the risk of cancer^14,15^. Moreover, the genetic sensitivity of the host can control the effects of the disease through complex interactions with environmental factors, and diets containing anticancer nutrients and carcinogens can modulate the risk of cancer development, especially in genetically vulnerable individuals^16,17^. Thus, gene-diet interactions can account for various outcome differences in individual GC risk, complementing the findings of studies investigating a single gene or a single diet.

In this study, we found that dietary inflammation is associated with the risk of GC. We also analyzed whether a *TNF* genetic polymorphism is related to the risk of GC. In particular, we focus on identifying the association between GC and a genetic polymorphism of *TNF* (rs1799964); the association between rs1799964 and both GC and *H. pylori* infection has been previously reported^18,19^. We aimed to study whether differences in individual *TNF* genes have different effects on GC depending on eating habits and vice versa.

## Results

### Study population characteristics

Table 1 presents the general characteristics of the study participants. The subjects with GC were more likely to be smokers and less likely to engage in regular exercise than the controls (p < 0.001 for all variables). In addition, the cases had lower education levels (p < 0.001) than the controls. The prevalence of *H. pylori* infection was higher among the cases (84.5%) than the controls (40.4%). These variables might be associated with the GC risk and, therefore, were included in the subsequent statistical analyses that considered potential confounders. The total energy intake and DII score of the cases were significantly higher than those of the controls (p < 0.001). However, no differences were found in the distribution of a family history of GC or alcohol consumption between the cases and controls. Additionally, regarding age and sex, there was no difference because the participants were matched beforehand.

**Table 1:** General characteristics of the study participants stratified by cancer status.

Category	Control (N = 752)	Case (N = 373)	P*
**Age**	53.8 ± 9.12	53.9 ± 9.39	0.92
**Sex (%)**			
Men	487 (64.8)	242 (64.8)	0.96
Women	265 (35.2)	131 (35.2)	
**Smoking status (%)**			
Non	339 (45)	148 (39.7)	< 0.001
Ex-smoker	254 (33.8)	107 (28.7)	
Current	159 (21.2)	117 (31.4)	
**Drinking status (%)**			
Non-current	274 (36.4)	148 (39.7)	0.2
Current	478 (63.6)	225 (60.3)	
**Education (%)**			
Element	47 (6.3)	57 (15.3)	< 0.001
Mid-high	353 (46.8)	233 (62.5)	
University	352 (46.8)	83 (22.3)	
**Regular exercise (%)**			
No	337 (44.8)	240 (64.3)	< 0.001
Yes	415 (55.2)	133 (35.7)	
***H. pylori infection (%)***			
Negative	448 (59.6)	58 (15.5)	< 0.001
Positive	304 (40.4)	315 (84.5)	
**First-degree family history of cancer (%)**			
Yes	367 (49.2)	176 (47.5)	0.8
No	383 (50.8)	197 (52.5)	
**Total energy intake**	1717.0 ± 549.1	1929.0 ± 662.0	< 0.001
**DII score**	0.64 ± 2.63	1.22 ± 2.59	< 0.001

The values in the table indicate the mean ± SD or N (%).

*P-values denote the difference between the cases and controls at the 95% confidence level.

### Association between the DII score and risk of GC

Table 2 presents the associations between the tertile ranges of the DII score and GC risk. To confirm the association between the DII score and GC, Model 1 was adjusted for potential confounding factors, such as regular exercise, education and smoking status, while Model 2 included the *H. pylori* infection status along with the variables included in Model 1. In the crude model, the participants in the highest DII group had a 1.70-fold higher GC risk than those in the lowest DII group (odds ratios (OR), 1.70; 95% confidence intervals (CI) 1.24–2.33). This association was also significant after adjusting for all confounding factors (Model 2: OR, 1.41; 95% CI 1.00–2.06). When stratified by sex, compared to the risk associated with the lowest DII score group, the women with the highest DII score showed an association with an increased risk of GC, but there was no significant association among the men (Model 1: OR, 1.99; 95% CI 1.15–3.50 for women, OR,1.04; 95% CI 0.69–1.57 for men; Model 2: OR, 2.27; 95% CI 1.25–4.19 for women, OR, 1.06; 95% CI 0.63–1.78 for men). The women also showed a trend of significantly increased GC risk as the DII increased (p-value for trend = 0.007, 0.004 in Model 1, Model 2, respectively). Additionally, sex showed a significant interaction with the DII in the context of the GC risk (p for interaction = 0.014). Stratified by the *H. pylori* infection status, the association between the DII and GC was observed only in the *H. pylori*-positive group (fully adjusted model: OR, 1.62; 95% CI 1.04–2.45), but the interaction between the DII and infection status was not significant (p = 0.36) (Supplementary Table S2).

**Table 2:** Associations between the dietary inflammatory index (DII) score and gastric cancer risk.

DII	N (%)		OR (95% CI)			P interaction
	Control	Case	Crude	Model 1	Model 2	
**Total**						
T1	251 (33.38)	91 (24.40)	1	1	1	
T2	251 (33.38)	128 (34.32)	1.41 (1.02–1.94)	1.36 (0.97–1.90)	1.39 (0.96–2.02)	
T3	250 (33.24)	154 (41.28)	1.70 (1.24–2.33)	1.38 (0.99 –1.93)	1.41 (1.00–2.06)	
P trend				0.06	0.07	
**Men**						0.014
T1	163 (33.48)	68 (28.1)	1	1	1	
T2	162 (33.26)	88 (36.36)	1.19 (0.80–1.79)	1.34 (0.89–2.03)	1.25 (0.79–1.96)	
T3	162 (33.26)	86 (35.54)	1.29(0.87–1.93)	1.04 (0.69–1.57)	0.99 (0.63–1.58)	
P trend				0.76	0.9	
**Women**						
T1	89 (33.58)	28 (21.4)	1	1	1	
T2	88 (33.21)	34 (25.9)	1.23 (0.69–2.21)	1.07 (0.58–1.97)	1.37 (0.71–2.65)	
T3	88 (33.21)	69 (52.7)	2.49 (1.48–4.28)	1.99 (1.15–3.50)	2.27 (1.25–4.19)	
P trend				0.007	0.004	

Model 1: adjusted by smoking status, education, and regular exercise for the total and men and adjusted by education and regular exercise for the women. Model 2: adjusted by smoking status, education, regular exercise, and *Helicobacter pylori* infection for the total and men and adjusted by education, regular exercise and *Helicobacter pylori* infection for the women. Criteria for DII: in total, T1 < − 0.77, − 0.77 < T2 < 2.01, T3 > 2.01; in men, T1 < − 0.17, − 0.17 < T2 < 2.93, T3 > 2.93; in women, T1 < − 1.59, − 1.59 < T2 < 1.41, T3 > 1.41. P interaction is the p-value of the interaction between sex and the DII.

OR, odds ratio; 95% CI, 95% confidence interval.

### Association between a *TNF* polymorphism (rs1799964) and the risk of GC

The results of the association between a genetic polymorphism of *TNF* (rs1799964) and the GC risk are shown in Table 3. Rs1799964 was not associated with the risk of GC (Model 1: OR, 1.08; 95% CI 0.84–1.41, Model 2: OR, 1.07; 95% CI 0.80–1.43).

**Table 3:** Associations between tumor necrosis factor (*TNF*) genetic polymorphisms and the gastric cancer risk in the dominant model.

*Gene (rs ID)*	Allele	N (%)		OR (95% CI)		
		Control	Case	Crude	Model 1	Model 2
**Total (N = 1,125)**						
*TNF (rs1799964)*	TT	475 (63.2)	230 (61.7)	1	1	1
	C+	277 (36.8)	143 (38.2)	1.07 (0.82–1.38)	1.08 (0.84–1.41)	1.07 (0.80–1.43)
**Men (N = 729)**						
*TNF (rs1799964)*	TT	301 (61.8)	154 (63.6)	1	1	1
	C+	186 (38.2)	88 (36.4)	0.92 (0.67–1.27)	1.01(0.72–1.42)	0.94 (0.64–1.38)
**Women (N = 396)**						
*TNF (rs1799964)*	TT	174 (65.7)	76 (58.0)	1	1	1
	C+	91 (34.3)	55 (42.0)	1.38 (0.90–2.13)	1.44 (0.91–2.25)	1.72 (0.96–2.62)

Model 1: adjusted by smoking status, education, and regular exercise for the total and men and adjusted by education and regular exercise for the women. Model 2: adjusted by smoking status, education, regular exercise, and *Helicobacter pylori* infection status for the total and men and adjusted by education, regular exercise and *Helicobacter pylori* infection status for the women. The values in the table indicate N (%), OR (odds ratios) and 95% CI (95% Confidence Intervals).

### Modifying effects of the *TNF* gene on the associations between the DII and GC risk

To identify whether the *TNF* gene modifies the association between the DII and GC risk, we performed a stratified analysis by individual genotype (Table 4). Compared to the risk in the low DII group, the *TNF* rs1799964 TT homozygotes in the high DII group showed a 1.62-fold increase in the GC risk (Model 1: OR, 1.62; 95% CI 1.16–2.28). However, with C, there was no significant association between the DII and GC risk, although the interaction effect was suggestive (p for interaction = 0.055). A significant interaction was observed among the women. Among the women, the TT homozygotes of rs1799964 in the high DII group exhibited an increased GC risk by 2.30 times compared to the risk in the low DII group (Model 1: OR, 2.30; 95% CI 1.27–4.24). However, among the C carriers, there was no statistical association between the DII and GC, although there was a decrease in the GC risk in the high DII groups (Model 1: OR, 0.78; 95% CI 0.37–1.65, p for interaction = 0.035). When we further adjusted for the *H. pylori* infection status in Model 2, a significant association between high DII scores and the GC risk was also observed only in the TT genotype carriers, although the statistical significance of the interaction disappeared (p for interaction = 0.068). In the case of TT genotype, an inflammatory diet increased the risk of GC by nearly more than 2 times compared with the risk of the C carrier group when comparing the high and low DII groups (Model 2: OR, 2.55; 95% CI 1.36–4.92 for TT genotype, OR, 0.96; 95% CI 0.40–2.26 for C carrier). Among the men, a significant interaction between the DII and *TNF* gene polymorphism could not be identified (Model 1: OR, 1.08; 95% CI 0.71–1.64 for TT genotype; OR, 1.09; 95% CI 0.63–1.90 for C carrier, Model 2: OR, 1.03; 95% CI 0.64–1.65 for TT genotype; OR, 1.17; 95% CI 0.64–2.14 for C carrier). Stratified by the *H. pylori* infection status, the same results were observed in the *H. pylori*-positive group. The TT homozygotes showed an increased risk of GC in the high DII group (fully adjusted model: OR, 1.76; 95% CI 1.13–2.73). However, among the individuals with the C allele, the risk of GC in the high DII group was decreased, although the association with DII and GC was not significant (OR, 0.98; 95% CI 0.54–1.77, p for interaction = 0.034). But, no association with DII and GC or an interaction effect was observed in the *H. pylori*-negative group (Table 5).

**Table 4:** Interaction of the *TNF* genetic polymorphism (dominant model) and DII score with the gastric cancer risk.

*Gene (rs ID)*	Allele	DII	N (%)		Crude	P interaction	Model 1	P interaction	Model 2	P interaction
			Control	Case	OR (95% CI)		OR (95% CI)		OR (95% CI)	
**Total (N = 1,125)**										
*TNF (rs1799964)*	TT	Low	244 (51.4)	85 (37.0)	1	0.14	1	0.055	1	0.12
		High	231 (48.6)	145 (63.0)	1.80 (1.31–2.49)		1.62 (1.16–2.28)		1.59 (1.10–2.31)	
	C+	Low	132 (47.7)	61 (42.7)	1		1		1	
		High	145 (52.3)	82 (57.3)	1.22 (0.82–1.84)		1.00 (0.63–1.52)		1.06 (0.66–1.75)	
**Men (N = 729)**										
*TNF (rs1799964)*	TT	Low	147 (48.8)	70 (45.5)	1	0.83	1	0.98	1	0.89
		High	154 (51.2)	84 (54.5)	1.21 (0.82–1.79)		1.08 (0.71–1.64)		1.03 (0.64–1.65)	
	C+	Low	85 (45.7)	36 (40.9)	1		1		1	
		High	101 (54.3)	52 (59.1)	1.29 (0.78–2.16)		1.09 (0.63–1.90)		1.17 (0.64–2.14)	
**Women (N = 396)**										
*TNF (rs1799964)*	TT	Low	92 (52.9)	22 (28.9)	1	0.042	1	0.035	1	0.068
		High	82 (47.1)	54 (71.1)	2.75 (1.56–4.98)		2.30 (1.27–4.24)		2.55 (1.36–4.92)	
	C+	Low	40 (44)	23 (41.7)	1		1		1	
		High	51 (56)	32 (58.2)	1.09 (0.56–2.16)		0.78 (0.37–1.65)		0.96 (0.40–2.26)	

Model 1: adjusted by smoking status, education, and regular exercise for the total and men and adjusted by education and regular exercise for the women. Model 2: adjusted by smoking status, education, regular exercise, and *Helicobacter pylori* infection status for the total and men and adjusted by education, regular exercise and *Helicobacter pylori* infection status for the women. Criteria for high and low DII groups: low < 0.724, high > 0.724 for total; low < 1.12, high > 1.12 for men; low < 0.038, high > 0.038 for women. The values in the table indicate N (%), OR (odds ratios) and 95% CI (95% Confidence Intervals). P interaction is the p-value of the interaction between the DII and *TNF* genotype.

OR, odds ratio; 95% CI, 95% confidence interval; *TNF*, tumor necrosis factor; DII, dietary inflammatory index.

**Table 5:** Interaction of the *TNF* genetic polymorphism (dominant model) and DII score with the gastric cancer risk stratified by *H. pylori* infection.

Infection status	*Gene (rsID)*	Allele	DII	N (%)		OR (95% CI)		P interaction
				Control (N = 304)	Case (N = 315)	Crude	Fully adjusted	
*H. pylori* positive	*TNF (rs1799964)*	TT	Low	99 (52.1)	68 (35.4)	1	1	0.034
			High	91 (47.9)	124 (64.6)	1.98 (1.32–2.99)	1.76 (1.13–2.73)	
		C+	Low	53 (46.5)	54 (43.9)	1	1	
			High	61 (53.5)	69 (56.1)	1.11 (0.67–1.85)	1.01 (0.54–1.77)	

Infection status	*Gene (rsID)*	Allele	DII	N (%)		OR (95% CI)		P interaction
				Control (N = 448)	Case (N = 58)	Crude	Fully adjusted	
*H. pylori* negative	*TNF (rs1799964)*	TT	Low	145 (50.9)	16 (42.1)	1	1	0.83
			High	140 (49.1)	22 (57.9)	1.36 (0.68–2.75)	1.38 (0.68–2.87)	
		C+	Low	79 (48.5)	7 (35.0)	1	1	
			High	84 (51.5)	13 (65.0)	1.75 (0.68–4.86)	1.25 (0.44–3.74)	

Fully adjusted model was adjusted by age, smoking status, education, and regular exercise. The values in the table indicate N (%), OR (odds ratios) and 95% CI (95% Confidence Intervals). Criteria for high and low DII groups: in *H. pylori* positive: low < 0.707, high > 0.707; in *H. pylori* negative: low < 0.742, high > 0.742. P interaction is the p-value of the interaction between the DII and *TNF* genotype.

OR, odds ratio; 95% CI, 95% confidence interval; *TNF*, tumor necrosis factor; DII, dietary inflammatory index.

## Discussion

In the present case–control study of 1,125 participants (373 cases and 752 controls), we observed a significant association between the DII and risk of GC in women but not men. We also identified significant modification effects on the association between the DII and risk of GC according to a *TNF* genetic polymorphism. Among the individuals with high DII scores, C alleles in rs1799964 showed a protective effect against eating habits associated with GC risks compared to the effects observed in individuals with different genotypes.

Chronic inflammation can contribute to the development of cancer through an inflammatory mediator, such as cytokines (e.g., TNF-α and IL) secreted by immune cells, leading to changes in the epigenome^20^. Although many GC studies focus on *H. pylori* infection, environment-induced inflammatory reactions or genetic differences can also increase the risk of GC. Among such environmental factors, we focused on diet factors considered to induce inflammation through oxidative stress reactions, and we could confirm that an inflammatory-inducing eating habit is more dangerous in women, which is consistent with previous research results^21,22^. These results may be attributed to differences in eating habits between men and women and the effects of sex hormones. According to previous studies investigating sex differences in the immune response, inflammation-related genes can be overexpressed in women due to differences in hormone conditions and rare genes on X chromosomes, and this overexpression results in poor prognoses among women suffering from chronic inflammatory diseases^23,24^. Additionally, in the present study, the men and women had different drinking habits. In the case of the women, 43% were current alcohol consumers, whereas 73% of the men were current alcohol consumers (Supplementary Table S1). Therefore, the results of the association may have differed according to sex.

Another factor related to the inflammatory response is the genetic predisposition of the host. In particular, single nucleotide polymorphisms (SNPs) present in the promoter of *TNF* have been shown to contribute to differences in individual inflammatory reactions^3,5,25^. Sugimoto et al.^19^ reported that rs1799964 C alleles were involved in the susceptibility to GC and can induce higher *TNF* production, especially in East Asian individuals. Additionally, rs1799964 T>C polymorphisms might be associated with a reduced risk of *H. pylori* infection^18^. Therefore, the association with GC risk in that region was expected, and a previous meta-analysis suggested the need for further studies to provide evidence supporting the association between *TNF*-1031 (rs1799964) and GC^8,9^, but such an association was not confirmed in our study. However, interestingly, according to our results, the effects of an inflammatory diet on the GC risk differ depending on the *TNF* rs1799964 genotype. The exact mechanism of this phenomenon has not been explained, but it is possible that the variation in the promoter region is related to gene expression^6^.

*TNF*-α is a multifunctional cytokine produced by monocytes and macrophages that plays an important role in promoting the inflammatory response and cell proliferation and inducing apoptosis^6^. Polymorphisms located in the promoter region of the *TNF* gene affect the transcription of theses genes^6^. Some studies have reported a correlation between polymorphism rs1799964 and the *TNF* mRNA expression level^26,27^. Nourian et al*.*^28^ demonstrated that the level of *TNF* mRNA expression was reduced in individuals with the genotype TT compared with that in individuals with other genotypes. Considering the mechanism by which the substances contained in the diet induce the secretion of cytokines and cause the inflammatory cell response^29^, we assume that if cytokine secretion is genetically altered, it can be difficult to identify the same influence across different genetic types.

Our results suggest that inflammation-inducing eating habits can result in different outcomes depending on the individual's genetic background, and it is believed that dietary interventions considering individual genotypes could help prevent GC early on. In addition, when we conducted further stratification analysis by the *H. pylori* infection status, the results suggested that these risky diet patterns can cause different cancer development depending on the individual's genetic background. However, the interpretation requires caution, and studies involving larger samples are warranted because the number of cases in the H. pylori-negative group was very small. Additionally, other types of cytokines, such as the interleukin (IL) family (e.g., IL-17 and IL-6), contribute to the inflammatory response^30,31^. Indeed, many studies have reported abnormal IL concentrations in GC patients and suggested promising indicators of GC development^32,33^. Therefore, further studies are needed to identify whether genetic polymorphisms associated with other cytokines can modify the diet effect that increases the risk of cancer.

To the best of our knowledge, this study is the first to report a gene-diet interaction while particularly focusing on a gene functionally associated with secreted serum *TNF*. Another strength of this study is that we calculated an index score to reflect the inflammatory potential of a diet, assessed its association with the risk of GC and used it to examine interactions with genes, which can consider the overall intake conditions and an individual’s genetic background rather than only one risk factor. However, our study has several limitations. First, because of the relatively small number of GC cases, we have insufficient statistical power. Second, although we had information regarding the cardiac or noncardiac cancer status of the GC cases, because the number of cases was small, we were unable to observe a difference in the GC risk according to cancer subtypes. Additionally, this study may have been affected by selection bias due to its hospital-based case–control study design.

In conclusion, our findings demonstrate that the inflammatory potential of diets is associated with an increase in the risk of GC, particularly among women. Although there was no evidence of an association between the *TNF* polymorphism and GC, rs1799964 may modulate the effect of diet on GC, and a pro-inflammatory diet had a greater effect in the general population who did not have a genetic variant. Therefore, these results may support dietary recommendations for GC prevention and therapy on the basis of gene-environment interactions. To prevent GC, it seems important to reduce the amount of food-induced inflammation and consume a diet reportedly associated with anti-inflammation, such as fruits and vegetables. Further evaluations of the associations of the dietary factors included in the DII calculation and studies of other cytokine genetic polymorphisms are necessary to better understand the heterogeneity in the etiology of GC.

## Methods

### Study population

**Figure 1:**
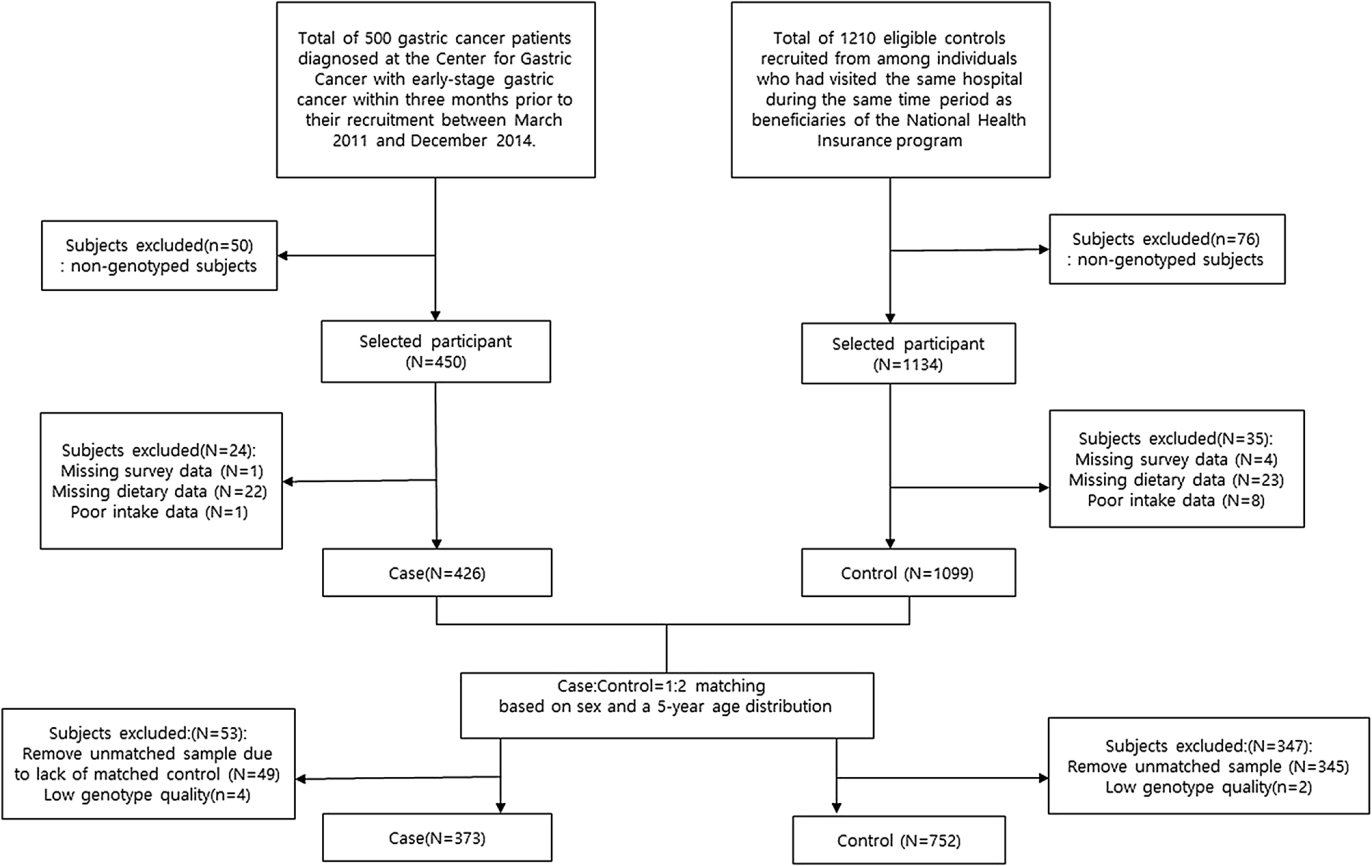
Flowchart of the selection of the study subjects in the study.

The participants were recruited to participate in a GC research project by the National Cancer Center (NCC) in Korea between March 2011 and December 2014. The details of this study have been previously described^34^. In brief, cases who were diagnosed with GC 3 months before recruitment were defined as patients. Cases diagnosed with other cancers within 5 years, diabetes mellitus, or severe mental disease and pregnant women were excluded. The controls were selected among individuals who visited the Center for Cancer Prevention and Detection at the NCC, and subjects with a history of cancer, diabetes mellitus, gastric ulcers, and *H. pylori* treatment were excluded. The cases and controls were matched for sex and 5-year age distributions. In total, 373 GC cases and 752 healthy controls were included in this study, and their genotype and dietary intake data were available (Fig. 1). All participants provided written informed consent, and the study protocol was approved by the Institutional Review Board of the NCC (IRB Number: NCCNCS-11-438). Additionally, all procedures used in this study were carried out in accordance with the guidelines of the Institutional Review Board of the NCC.

### Genotype measurement

The genotyping and quality control process were presented in detail in a previous study^35^. In brief, genomic DNA was extracted from peripheral blood, and the Affymetrix Axiom Exom 319 Array (Affymetrix Inc., Santa Clara, CA) platform, including 318,983 variants, was used for genotyping. Genotype imputation was performed using the Asian population (n = 504) in 1,000 Genome haplotypes phase III integrated variant set release GRch37/hg19 (https://www.1000genomes.org/) as a reference panel. Genetic markers with a minor allele frequency (MAF) < 0.01, call rate < 95%, and deviation from the Hardy–Weinberg equilibrium (*P*-value < 1 × 10^–6^) were discarded. SHAPIT (v2.r837) and IMPUTE2 (2.3.2) were used to perform the phasing and SNP imputation, respectively. The quality control criteria were applied after filtering for an INFO score over 0.6. Finally, the genetic polymorphism (rs1799964) in the *TNF* promoter was selected.

### Dietary assessment

#### Dietary assessment and calculation of the DII

The dietary intake of each participant was assessed using a 106-item semiquantitative food frequency questionnaire (SQFFQ). The validity and reproducibility of the SQFFQ have been previously reported^36^. The participants provided their individual average frequency of intake and portion sizes of specific foods in the year preceding the interview. These values were converted to obtain the daily nutrient intake using a scale with nine frequency categories (never or rarely, once a month, two or three times a month, once or twice a week, three or four times a week, five or six times a week, once a day, twice a day, and three times a day) and three portion size categories (small, medium, and large) included in the SQFFQ. The total energy and nutrient intake of each participant were analyzed by the Computer Aided Nutritional analysis program (CAN-PRO 4.0, Korean Nutrition Society, Seoul, Korea). The SQFFQ-derived data were used to calculate the DII scores of all participants. The details of the development^37^ and construct validation^38,39^ of the DII have been previously described. The dietary data were used to calculate the z-score of each food parameter for each individual. The global means and standard deviations of the food and nutrient intakes collected from 11 nations, including Korea, were used to calculate the z-score. Then, the z-score was converted to a percentile and centered by doubling the value and subtracting 1. The centered percentile value was multiplied by the respective inflammatory effect score to obtain the food parameter-specific DII score^37^. The overall DII score was calculated as the sum of all available food parameter-specific DII scores. This study included the following 35 of the original 45 DII food parameters to generate the DII score: protein, fat, carbohydrate, fiber, monounsaturated fatty acid (MUFA), polyunsaturated fatty acid (PUFA), saturated fatty acid, n-3 fatty acids, n-6 fatty acids, cholesterol, thiamin, riboflavin, niacin, vitamin B6, vitamin B12, vitamin C, folic acid, vitamin A, vitamin D, vitamin E, β-carotene, iron, magnesium, selenium, zinc, garlic, ginger, onion, green tea flavan-3-ols, flavones, flavonols, flavanones, anthocyanidins, and isoflavones. Energy was not used to compute the DII because all components of the DII were adjusted for energy intake using the energy density approach, which was calculated per 1,000 kcal of energy^40^. The world database was also standardized to 1,000 kcal/day.

### Statistical analyses

The difference in general characteristics between the cases and control subjects were analyzed by using the chi-square test (categorical variables) or t-test (continuous variables). The DII score was categorized into tertiles based on the control’s score to investigate the association between inflammatory intake and the GC risk. The OR and 95% CI of the GC risk were calculated for each group using a univariate logistic regression for the crude model and a multiple logistic regression with the lowest tertile as a reference group. The multivariable model (Model 1) was adjusted for age, sex, education level, smoking status, and physical activity. Model 2 additionally adjusted for the *H. pylori* infection status in Model 1. A stratified analysis by sex and *H. pylori* infection status was performed, and the median value of each DII tertile was used as a continuous variable to test for trends. Genetic association analyses were performed in the dominant models. To analyze the interaction effects between the DII score and the genetic polymorphism, low- and high-score groups were categorized according to the median DII score of the control group. The interaction between the DII and SNPs was analyzed by using the likelihood ratio test between the models with and without the interaction term (DII*SNPs). All statistical analyses were performed using PLINK version 1.07^41^ and R software version 3.4.3.

## Supplementary information

Supplementary Information 1.
