# Supplementary material for: TNF genetic polymorphism (rs1799964) may modify the effect of the dietary inflammatory index on gastric cancer in a case–control study
Source: Sci Rep. 2020 Sep 3;10:14590. doi: 10.1038/s41598-020-71433-9 (PMC7471946; doi:10.1038/s41598-020-71433-9)
Supplement: Supplementary file 1 — Supplementary Information 1. [file 41598_2020_71433_MOESM1_ESM.docx]

**Supplementary Information**

***TNF* genetic polymorphism (rs1799964) may modify the effect of the dietary inflammatory index on gastric cancer in a case-control study**

**Author names and affiliations:**

Jeeeun Kim^1,2^, Jeonghee Lee^1^, Il Ju Choi^3^, Young-Il Kim^3^, Joohon Sung^2^ and Jeongseon Kim^1^*

^1^ Department of Cancer Biomedical Science, Graduate School of Cancer Science and Policy, National Cancer Center, Goyang, South Korea

^2^ Division of Genome and Health Big Data, Department of Public Health Sciences Graduate School of Public Health, Seoul National University, Seoul, South Korea^.^

^3^ Center for Gastric Cancer, National Cancer Center, Goyang-si, South Korea

***Correspondence:**

Jeongseon Kim, PhD, Department of Cancer Biomedical Science, Graduate School of Cancer Science and Policy, National Cancer Center, Goyang-si, 10408, Gyeonggi-do, South Korea. Tel.: +82-31-920-2570; Fax: 82-31-920-2579; E-mail: [jskim@ncc.re.kr](mailto:jskim@ncc.re.kr)

**Supplementary Table S1** General characteristics of study participants stratified by sex

| Category |  | Men(N=729) | | | Women(N=396) | | |
| --- | --- | --- | --- | --- | --- | --- | --- |
|  |  | Control(N=487) | Case(N=242) | P* | Control(N=265) | Case(N=131) | P* |
| Age |  | 54.86 ±8.51 | 55.01±8.70 | 0.824 | 51.73 ± 9.86 | 51.85±10.28 | 0.908 |
| Smoking status (%) | Non | 100 (20.5) | 32 (13.2) | <0.001 | 239 (90.2) | 116 (88.5) | 0.767 |
|  | Ex-smoker | 244 (50.1) | 100 (41.3) |  | 10 (3.8) | 7 (5.3) |  |
|  | Current | 143 (29.4) | 110 (45.5) |  | 16 (6.0) | 8 (6.1) |  |
| Drinking status (%) | Non-current | 125 (25.7) | 72 (29.8) | 0.364 | 149 (56.2) | 76 (58.0) | 0.715 |
|  | Current | 362 (74.3) | 170 (70.2) |  | 116 (43.8) | 55 (42.0) |  |
| Education (%) | Element | 27 (5.5) | 32 (13.2) | <0.001 | 20 (7.5) | 25 (19.1) | <0.001 |
|  | Mid-high | 201 (41.3) | 155 (64.0) |  | 152 (57.4) | 78 (59.5) |  |
|  | University | 259 (53.2) | 55 (22.7) |  | 93 (35.1) | 28 (21.4) |  |
| Regular exercise (%) | No | 211 (43.3) | 144 (59.5) | <0.001 | 126 (47.5) | 96 (73.3) | <0.001 |
|  | Yes | 276 (56.7) | 98 (40.5) |  | 139 (52.5) | 35 (26.7) |  |
| *H. pylori* infection (%) | Negative | 298 (61.2) | 38 (15.7) | <0.001 | 150 (56.6) | 20 (15.3) | <0.001 |
|  | Positive | 189 (38.8) | 204 (84.3) |  | 115 (43.4) | 111 (84.7) |  |
| First degree family history of cancer (%) | Yes | 235 (48.3) | 113 (46.7) | 0.691 | 134 (50.6) | 63 (48.1) | 0.643 |
|  | No | 252 (51.7) | 129 (53.3) |  | 131 (49.4) | 68 (51.9) |  |
| Total energy intake |  | 1771.8 ± 555.1 | 2065.1 ± 705.6 | <0.001 | 1616.3 ± 524.1 | 1677.5 ± 482.3 | 0.263 |
| DII score |  | 0.97 ± 2.56 | 1.34 ±2.48 | 0.063 | 0.04 ± 2.66 | 1.00 ± 2.78 | 0.001 |

The values in the table indicate: Mean ± S.D. or n (%) * P-values denote the difference between cases and controls at the 95% confidence level

**Supplementary Table S2** Associations of dietary inflammatory index (DII) score with gastric cancer risk.

| Infection status | No. (%) |  | OR (95% CI) | | P interaction |
| --- | --- | --- | --- | --- | --- |
|  |  |  |  |  | 0.36 |
| *H. pylori* positive | Control(N=304) | Case(N=315) | Crude | Fully adjusted |  |
| T1 | 102(33.6) | 74(23.5) | 1 | 1 |  |
| T2 | 101(33.2) | 108(34.3) | 1.47(0.99-2.21) | 1.58(1.03 -2.48) |  |
| T3 | 101(33.2) | 133(42.2) | 1.82(1.22-2.70) | 1.62(1.04 -2.45) |  |
| P trend |  |  |  | 0.042 |  |
| *H. pylori* negative | Control(N=448) | Case(N=58) |  |  |  |
| T1 | 150(33.5) | 17(29.3) | 1 | 1 |  |
| T2 | 149(33.3) | 20(34.5) | 1.18(0.6-2.37) | 0.97(0.47-2.02) |  |
| T3 | 149(33.3) | 21(36.2) | 1.24(0.63-2.48) | 1.04(0.52-2.22) |  |
| P trend |  |  |  | 0.86 |  |

Fully adjusted model was adjusted by age, smoking status, education, and regular exercise. Criteria for DII groups: in *H. pylori* positive: T1<-0.79, -0.79<T2<2.01, T3>2.01 in *H. pylori* negative: T1<-0.71, -0.71 <T2<2.03, T3>2.03 The values in the table indicate: N(%) and OR (odds ratios) and 95% CI (95% Confidence Intervals) P interaction is the p-value for the interaction between infection status and DII
